# Non-invasive assessment of fluid responsiveness to guide fluid therapy in patients with sepsis in the emergency department: a prospective cohort study

**DOI:** 10.1136/emermed-2020-209771

**Published:** 2021-04-22

**Authors:** Nienke K Koopmans, Renate Stolmeijer, Ben C Sijtsma, Paul A van Beest, Christiaan E Boerma, Nic J Veeger, Ewoud ter Avest

**Affiliations:** 1 Emergency Medicine, Medical Centre Leeuwarden, Leeuwarden, The Netherlands; 2 Emergency Medicine, University Medical Centre Groningen, Groningen, The Netherlands; 3 Anesthesiology, Medical Centre Leeuwarden, Leeuwarden, The Netherlands; 4 Intensive Care Medicine, Medical Centre Leeuwarden, Leeuwarden, The Netherlands; 5 Epidemiology, Medical Centre Leeuwarden, Leeuwarden, The Netherlands; 6 Epidemiology, University of Groningen, University Medical Center Groningen, Groningen, The Netherlands; 7 Air Ambulance Kent, Surrey and Sussex, Redhill, UK

**Keywords:** infectious diseases, bacterial, non invasive, treatment, clinical management

## Abstract

**Background:**

Little is known about optimal fluid therapy for patients with sepsis without shock who present to the ED. In this study, we aimed to quantify the effect of a fluid challenge on non-invasively measured Cardiac Index (CI) in patients presenting with sepsis without shock.

**Methods:**

In a prospective cohort study, CI, stroke volume (SV) and systemic vascular resistance (SVR) were measured non-invasively in 30 patients presenting with sepsis without shock to the ED of a large teaching hospital in the Netherlands between May 2018 and March 2019 using the ClearSight system. After baseline measurements were performed, a passive leg raise (PLR) was done to simulate a fluid bolus. Measurements were then repeated 30, 60, 90 and 120 s after PLR. Finally, a standardised 500 mL NaCl 0.9% intravenous bolus was administered after which final measurements were done. Fluid responsiveness was defined as >15% increase in CI after a standardised fluid challenge.

**Measurements and main results:**

Seven out of 30 (23%) patients demonstrated a >15% increase in CI after PLR and after a 500 mL fluid bolus. Fluid responders had a higher estimated glomerular filtration rate (eGFR) (64 (44–78) vs 37 (23–47), p=0.009) but otherwise similar patient and treatment characteristics as non-responders. Baseline measurements of cardiac output (CO), CI, SV and SVR were unrelated to PLR fluid responsiveness. The change in CI after PLR was strongly positive correlated to the change in CI after a 500 mL NaCl 0.9% fluid bolus (r=0.88, p<0.001).

**Conclusion:**

The results of the present study demonstrate that in patients with sepsis in the absence of shock, three out of four patients do not demonstrate a clinically relevant increase in CI after a standardised fluid challenge. Non-invasive CO monitoring in combination with a PLR test has the potential to identify patients who might benefit from fluid resuscitation and may contribute to a better tailored treatment of these patients.

**Key messages**

**What is already known on this subject**

- Although most patients presenting with sepsis in the ED do not fulfil criteria for septic shock, intravenous fluid resuscitation is often initiated in these patients.
- Intravenous fluid administration has been shown to have positive on static haemodynamic parameters such as mean arterial pressure and heart rate have been reported, but evidence shows static measures are not related to outcome.
- Dynamic circulatory assessment such as the change in Cardiac Index would be preferable, but invasive monitoring is not appropriate in these patients.

**What this study adds**

- In this prospective study of 30 ED patients with sepsis, we used a non-invasive system to measure dynamic circulatory changes in patients with sepsis without shock in response to passive leg raise (PLR) and a fluid challenge.
- Only 23% of patients with sepsis in the absence of shock demonstrated a clinically relevant increase in Cardiac Index in response to either PLR or a fluid challenge.
- Non-invasive cardiac output monitoring in combination with a PLR test has the potential to identify fluid responsive patients and may contribute to more tailored treatment of sepsis.

## Introduction

Sepsis results in over 75 000 hospital admissions and over 15 000 deaths each year in the UK.^1^ The high disease burden and the perception that sepsis-associated death is preventable^2 3^ has resulted in the development of specific tools for early recognition and treatment of sepsis.^4 5^ For these patients with septic shock, early antibiotics and intravenous fluids are the cornerstones of their treatment, although massive crystalloid transfusion is no longer advocated.^6 7^ Invasive cardiac output (CO) measurement is often applied in these patients to acquire dynamic variables of fluid responsiveness such as changes in CO and Cardiac Index (CI) in response to fluid administration, in order to guide treatment.^8^

The majority of patients with sepsis presenting to the ED, however, do not fulfil the criteria for septic shock,^9^ and little is known about optimal fluid therapy for these patients. Previous studies have shown that intravenous fluids are often administered to patients with sepsis in the absence of organ failure or shock.^10^ The effect on dynamic circulatory parameters, however, is largely unknown, as invasive haemodynamic monitoring is usually not performed. Although positive effects of intravenous fluid administration on static haemodynamic parameters such as mean arterial pressure (MAP) and heart rate have been reported, it is well recognised that these static measures are not related to outcome.^11^ In recent years, however, non-invasive methods to measure CO reliably have become available. This provides the opportunity to monitor dynamic circulatory parameters in a broader category of patients.

In this study, we aimed to quantify the effect of a fluid challenge on non-invasively measured CI in patients presenting with sepsis without shock. Furthermore, we aimed to identify differences in patient or treatment characteristics between fluid responders and non-responders.

## Methods

### Study setting and design

We performed a single centre prospective study in patients with sepsis at presentation to the ED of the Medical Center Leeuwarden (a teaching hospital in the Netherlands with 27 000 ED visits yearly) between May 2018 and March 2019.

### Study population

Patients were eligible to participate if they presented to the ED with sepsis. In line with sepsis-3 guidelines, there had to be evidence of an infection (defined as a temperature <36°C or >38°C without an obvious cause for hypothermia or hyperthermia) in combination with evidence of organ dysfunction (defined as the presence of at least one of the quick sepsis-related organ failure assessment (qSOFA) criteria (altered mental state, hypotension (MAP <65 mm Hg or systolic blood pressure (SBP) <100 mm Hg) or a respiratory rate >22/min) or hypoxemia (oxygen saturation (SpO_2_) <94% or 5% lower than baseline) or a lactate >2 mmol/L in point of care arterial blood gas analysis or venous blood gas analysis performed directly at presentation). Other signs of organ dysfunction requiring continuous observation and/or lab results not immediately available at presentation (eg, acute renal dysfunction, coagulopathy, hyperbilirubinemia, decreased urinary output) were not used for sepsis definition (and thereby patient selection).

Patients could not participate when they were <16 years, when they had a (presumed) increased abdominal pressure (including pregnant women) or when they presented with a concurrent acute event requiring immediate medical, endovascular or surgical intervention (cerebral events, acute coronary syndrome, acute pulmonary oedema, status asthmaticus, active gastrointestinal bleeding or trauma). Patients with known metastatic cancer in a palliative setting were excluded in order to minimise length of stay for these patients in the ED.

Patients with septic shock (ie, those who remained hypotensive after fluid bolus administration or needing vasopressor therapy to obtain a MAP >65 mm Hg in the presence of a lactate >2 mmol/L) and subjects for whom it was impossible to measure CO reliably in a non-invasive manner were excluded.

### Non-invasive CO measurements

CO was measured non-invasively using the ClearSight system (Edwards Lifesciences, Irvine, California, USA). The methodology of the ClearSight is based on the pulsatile unloading of the finger arterial walls using an inflatable finger cuff with a built-in photoelectric plethysmograph that uses pressure to maintain a constant blood volume in the finger. ClearSight calculates beat-to-beat stroke volume (SV) by dividing the area under the SBP curve (measured at 200 Hz) by the aortic input impedance (Zin). The value of Zin is determined from a three-element Windkessel model in which the non-linear effect of MAP and the influence of the patient’s age, height, weight and gender on aortic mechanical properties are incorporated. Because the waveform at the finger shows a more undulatory appearance than the radial pressure waveform, the system transforms the finger waveform into a brachial waveform with a specific filter. ClearSight uses the integrated area under the pulsatile systolic waveform from the brachial pressure wave as an input to the model, which directly yields SV and produces CO by multiplying beat-to-beat SV by instantaneous heart rate. The Nexfin technology used in the ClearSight has been shown to, although not interchangeable with transpulmonary thermodilution, be convenient and consistent to continuously measure CI, and could track the direction of changes under dynamic conditions.^12 13^

### Study procedures

**Figure 1:**
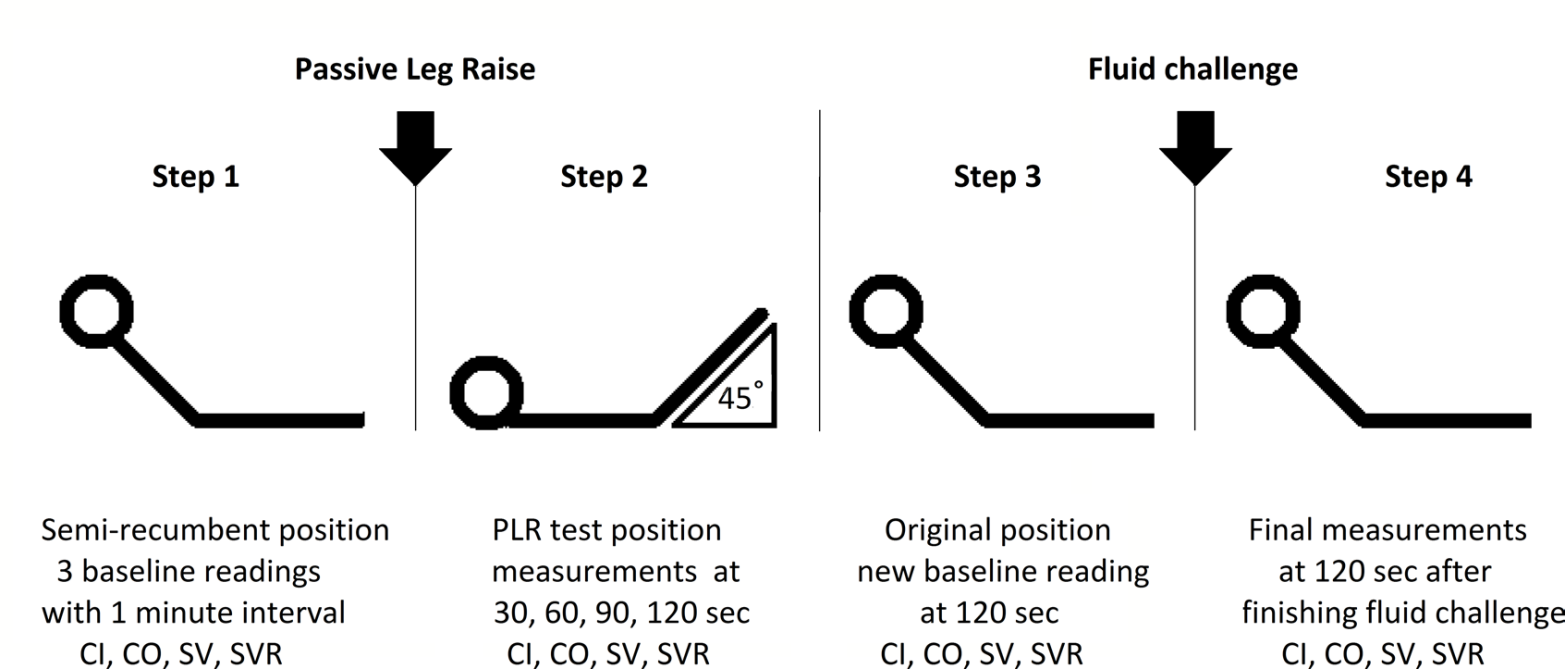
Overview of study procedures. CI, Cardiac Index; CO, cardiac output; PLR, passive leg raise; SV, stroke volume; SVR, systemic vascular resistance.

The finger cuff of the ClearSight system was applied to the patients finger as soon as possible after arrival in the ED and after consent was obtained, but no sooner than 5 min after transfer to the ED trolley. This was to minimise adrenergic stimulation by anxiety or pain. After the system was calibrated and a reliable signal was obtained, three baseline readings were done for CO, CI, SV and systemic vascular resistance (SVR), separated by 1 min intervals. Subsequently, a standardised passive leg raise (PLR) test was performed a to simulate a fluid bolus. The PLR test induces an auto transfusion of around 300 mL of fluids.^14^ Patients started in a semirecumbent position under 45°. The body is then moved in a full supine position with subsequent positioning of both legs raised at 45° from the bed for 120 s, and measurements of CO, CI, SV and SVR were repeated after 30, 60, 90 and 120 s via the ClearSight device (online supplemental figure 1). Thereafter, the patient was returned to his/her original position and after 120 s a new baseline reading was done. In accordance with literature,^15^ a cut-off value of 15% increase in CI after PLR was used to differentiate between fluid responders and non-responders. Finally, all patients were administered 500 mL NaCl 0.9% intravenous over a duration of 15 min. After finishing the fluid challenge for 120 s, CO, CI, SV and SVR were measured for a last time (figure 1).

10.1136/emermed-2020-209771.supp1 Supplementary data

### Data collection

The following parameters were collected on a dedicated case report form: patient demographics, prehospital data (oxygen and fluid administration, duration of symptoms, antibiotic treatment), vital parameters at presentation, (POCT)-lab results, presumed source of infection and non-invasive circulatory parameters at presentation, directly after PLR test and after a standardised fluid challenge. The sepsis warning and severity scores Systemic Inflammatory Response Score, Modified Early Warning Score (MEWS), National Early Warning Score (NEWS), qSOFA and Pneumonia Severity Score (CURB-65) scores were calculated based on information available at presentation, and patient disposition, patient re-disposition to intensive care unit within 48 hours and mortality within 30 days were extracted from the hospital’s electronic patient record (EPIC).

### Outcomes

The primary outcome was defined as the percentage of subjects in whom a standardised simulated fluid bolus by a PLR test resulted in a 15% or more improvement in non-invasively measured CI.

Secondary outcomes were defined as the relation between the changes in CI after respectively PLR and a standardised fluid bolus of 500 mL NaCL 0.9%, the relation between baseline CI, CO, SVR and SV with the change in CI after respectively PLR and a standardised fluid bolus and as the difference in patient or treatment characteristics between fluid responders and non-responders.

### Sample size

Sample size was calculated based on CO, as no prior trials were conducted with CI as primary outcome measure in similar populations. As CI is more frequently used than CO in clinical practice, we choose CI as our primary endpoint. Normal CO is variable between 4.0 and 8.0 L/min.^16^ Assuming a mean CO of 5.5 L/min, we estimated 30 patients were needed to detect a (clinically relevant) difference of 0.8±1.4 L/min (15% increase) after a pre-load modifying manoeuvre with a power of 90% and an alpha of 0.05.^17^ Accounting for a 10% attrition rate, it was decided to recruit 35 patients.

### Data analysis

Patient and treatment characteristics (including CO, CI, SV and SVR) are presented as median (IQR) or counts (%). Changes in CI in response to PLR or fluid administration were assessed using paired t-test (described as means (95% CI)) or Wilcoxon signed rank test (described as medians (IQR), whichever was appropriate. When comparing patient characteristics and responses of fluid responders versus non-responders, a Mann-Whitney U test and (exact) χ^2^ or Fisher’s exact test was used, where appropriate. Univariate correlation coefficients for CI, CO, SV and SVR after PLR and after fluid bolus administration were calculated. Missing data were reported in the Results section according to the Strengthening the Reporting of Observational Studies in Epidemiology guideline.^18^ A p value <0.05 was considered statistically significant. All statistical analyses were conducted with SAS software, V.9.4 (SAS Institute).

Pre-planned sensitivity analysis based on prehospital fluid administration was performed. Additionally, a pre-planned sensitivity analysis was performed assuming that the excluded patients were either all fluid responders or non-responders.

### Patient and public involvement

This research was done without patient involvement. Patients were not invited to comment on the study design and were not consulted to develop patient relevant outcomes or interpret the results. Patients were not invited to contribute to the writing or editing of this document for readability or accuracy.

## Results

### Study population

**Figure 2:**
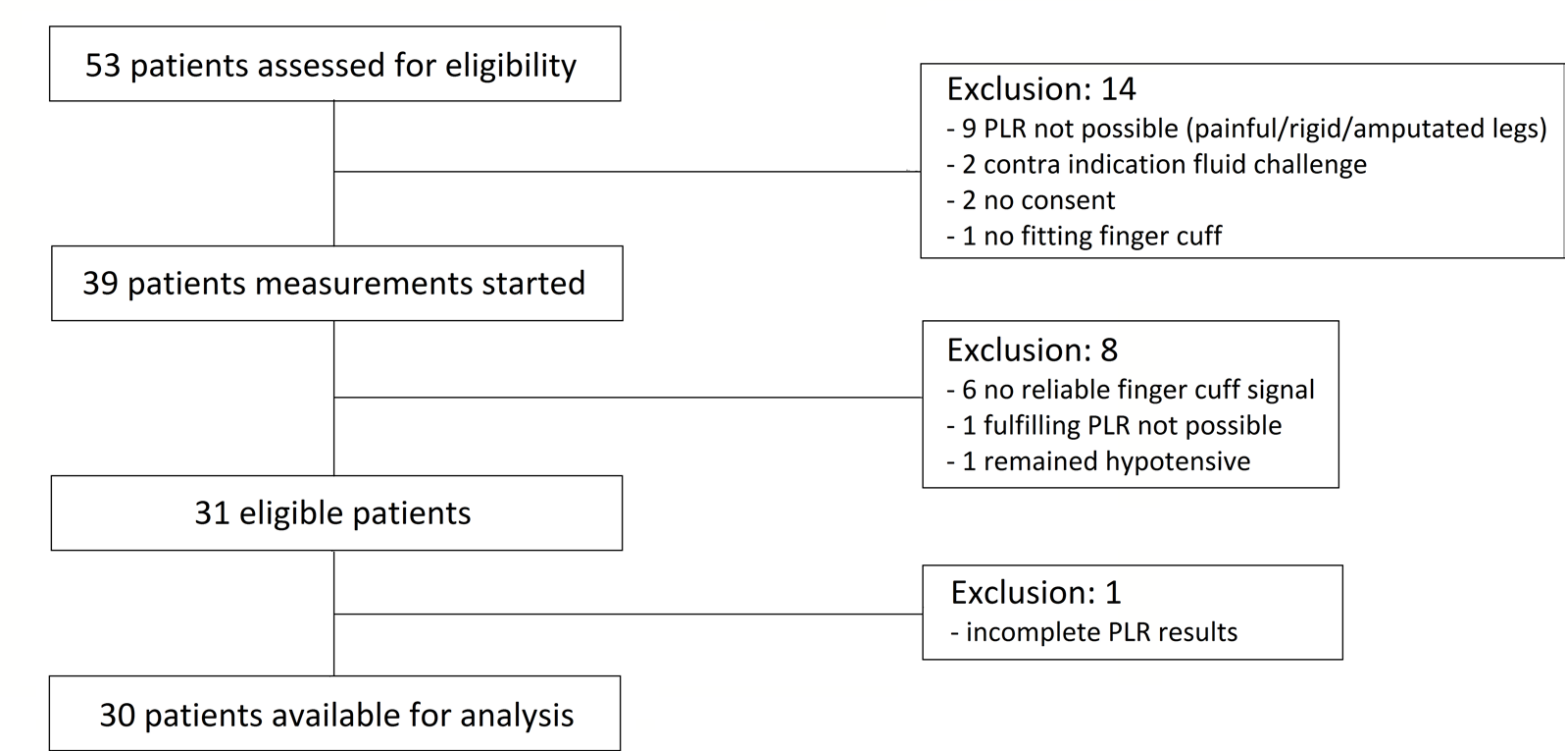
Patient inclusion. PLR, passive leg raise.

During the study period, 53 ED patients with sepsis were screened for inclusion. In nine patients, PLR was not possible due to painful or amputated legs, two patients were on dialysis or had end stage heart failure precluding the administration of a fluid bolus, two patients did not give consent and one patient did not fit the Clearsight finger cuffs. Ultimately, 39 patients were enrolled. In six patients, a reliable finger cuff signal could not be obtained. One patient could not complete the PLR test, and one patient remained hypotensive after initial resuscitation with 2000 mL crystalloids and was excluded under the presumption of septic shock (figure 2). One patient had to be excluded after completion of the measurements due to incomplete PLR results (missing t=30 s measurement), leaving 30 patients for the final analysis of the primary endpoint.

Median age of the patients was 75 (68–83) years. The majority of patients had a pulmonary (n=12) or urological (n=14) source of their infection. Seven (23%) of the patients were treated with antibiotics before arrival in the ED, 13 (43%) patients received oxygen and four patients (14%) had received intravenous fluids (mean 375 mL). Other patient characteristics and vital signs on ED arrival are represented in table 1.

**Table 1:** Study population characteristics of patients presenting in the emergency department with uncomplicated sepsis (n=30)

Median (IQR) or n (%)	
*Demographics*	
Gender (male) (n,%)	18 (60)
Age (years)	75 (68–83)
Length (cm)	173 (167–180)
Weight (kg)	77 (70–86)
*Prehospital*	
Duration of symptoms (days)	1 (1–3)
Antibiotic therapy (n,%)	7 (23.3)
Fluid therapy (n,%)*	4 (13.8)
Amount of fluid (mL)	375 (200–500)
Oxygen therapy (n,%)	13 (43.4)
*Vital signs at ED presentation*	
Heart rate (bpm)	110 (100–115)
SBP (mm Hg)	130 (117–153)
MAP (mm Hg)	90 (78–108)
SpO_2_ (%)	94 (93–95)
RR (/min)	25 (22–30)
Temperature (°C)	39.2 (38.5–39.6)
AVPU (n,%)	
A	24 (80)
V	6 (20)
P/U	0 (0)
GCS (n,%)	15 (14–15)
Confusion present (n,%)	11 (36.7)
*Sepsis warning and severity scores*	
SIRS	4 (3–4)
NEWS	8 (7–9)
MEWS	5 (4–7)
qSOFA	1 (1–2)
CURB-65	2 (1–3)

*Missing data for one patient regarding prehospital fluid therapy.

AVPU, Alert-Verbal-Painful-Unresponsive; CURB-65, Pneumonia Severity Score; MAP, mean arterial pressure; MEWS, Modified Early Warning Score; NEWS, National Early Warning Score; qSOFA, quick sepsis-related organ failure assessment; SBP, systolic blood pressure; SIRS, Systemic Inflammatory Response Score; SpO_2_, oxygen saturation.

### Analysis of outcome measures

#### Fluid responsiveness after PLR and fluid bolus

**Figure 3:**
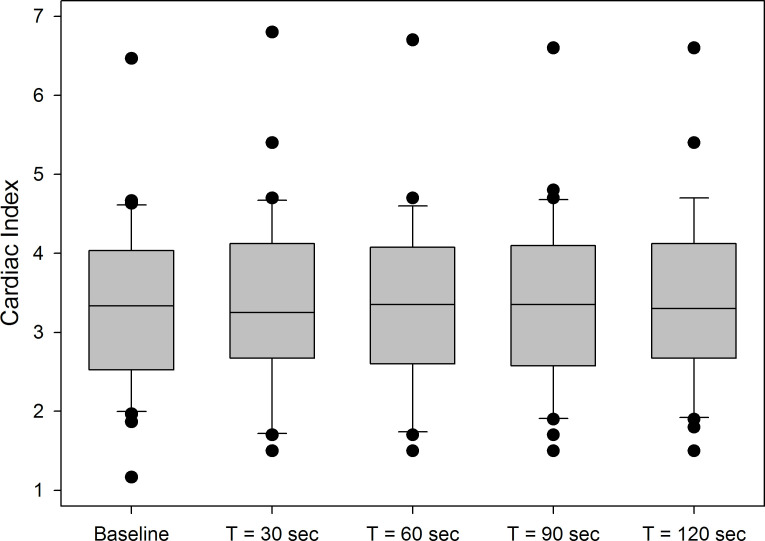
Boxplots of Cardiac Index at baseline and 30 s, 60 s, 90 s and 120 s after PLR (n=30). PLR, passive leg raise.

Median (IQR) CI was 3.3 L (2.5–4.0)/min/m^2^ at baseline. For most patients, CI peaked 30 s after the start of the PLR procedure (figure 3). Median maximum CI was 3.4 (2.8–4.2) L/min/m^2^, (mean (95% CI) change compared with baseline 0.22 (0.09–0.36) L/min/m^2^, p=0.003). PLR resulted in a 15% or greater increment in CI in 7/30 (23%) of the patients. Median maximum CI after the 500 mL NaCl 0.9% fluid challenge was 3.3 (2.8–3.9) L/min/m^2^ (mean (95% CI) change 0.12 (−0.36 to 0.28) L/min/m^2^, p=0.127). As with PLR, the fluid bolus resulted in a >15% increase in CI in 7/30 (23.3%) of the patients.

#### Sensitivity analyses

Four patients received prehospital fluids, all of whom were fluid non-responders (online supplemental table 1). Sensitivity analysis including only patients who had not received any prehospital fluid (n=26) did not change the primary outcome: (baseline CI 3.3 (2.5–3.9) L/min/m^2^, 3.3 (2.8–4.1) L/min/m^2^ after PLR (average change 0.22±0.39 L/min/m^2^, p=0.009) and 3.3 (2.8–3.8) L/min/m^2^ (average change 0.12±0.43 L/min/m^2^, p=0.17) after fluid bolus administration).

10.1136/emermed-2020-209771.supp2 Supplementary data

Sensitivity analysis including enrolled but subsequently excluded patients (n=9, total n=39) demonstrated that the percentage of patients with a >15% increment in CI after PLR or fluid bolus would be 17.9% if all were non-responders and maximally 41.0% if all were fluid responders.

#### Patient, treatment and disease characteristics in relation to fluid responsiveness

Patients responding with a more than 15% increase in CI after PLR (responders) were older (82 (73–85) vs 73 (68–83) years, p=0.19), had a lower heart rate (96 (91–115) vs 110 (103–115) bpm, p=0.28), worse renal function (with increased creatinine (165 (102–185) vs 98 (74–136) µmol/L, p=0.07) and BUN concentrations (12.5 (8.9–14.4) vs 7.8 (5.9–11.9) mmol/L, p=0.07)) although none of these comparisons was statistically significant. However, estimated Glomerular Filtration Rate (eGFR) was significantly higher in non-responders (64 (44–78) vs 37 (23–47), p=0.009). There was no difference in sepsis severity (as quantified with NEWS, MEWS and qSOFA scores) and patient disposition between responders and non-responders (see online supplemental table 1 for a full list of patient, treatment and disease characteristics).

#### Association of PLR and fluid bolus with changes in CI

**Figure 4:**
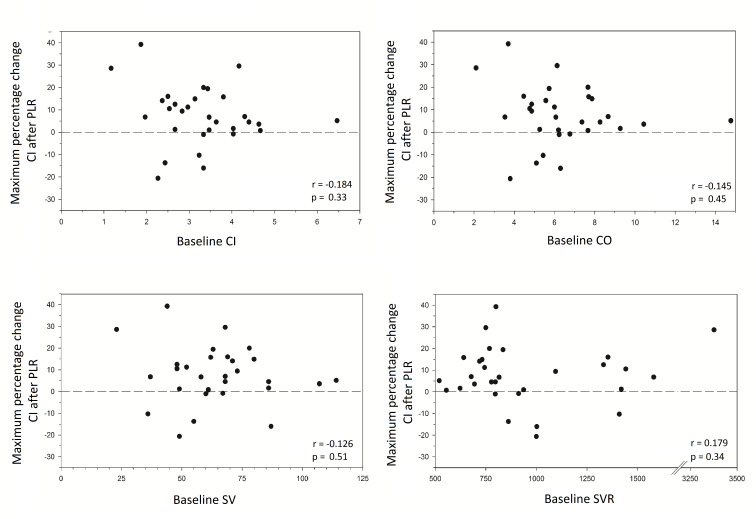
Scatterplots of maximum change in Cardiac Index after passive leg raise versus baseline cardiac output, Cardiac Index, stroke volume and systemic vascular resistance (n=30). CI, Cardiac Index; CO, cardiac output; PLR, passive leg raise; SV, stroke volume; SVR, systemic vascular resistance.

**Figure 5:**
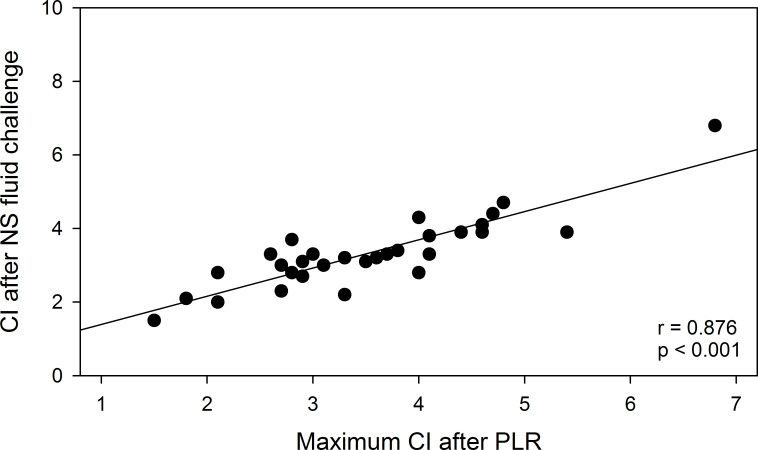
Scatterplot of paired data of individual patients of Cardiac Index after passive leg raise versus Cardiac Index after administration of a standardised intravenous fluid bolus of 500 mL NaCl 0.9% in ED patients with uncomplicated sepsis (n=30). CI, Cardiac Index; ED, emergency department; NS, NaCl 0.9%; PLR, passive leg raise test.

Table 2 shows baseline values of CI, SV and SVR at presentation in the ED. None of these non-invasively measured haemodynamic variables were significantly different at baseline between patients responding to a PLR with a >15% increase in their CI compared with those who did not. Baseline values of CI, SV and SVR were also unrelated to the maximum percentage change in CI after PLR (figure 4). However, non-invasively measured CI after administration of 500 mL NaCl 0.9% was highly correlated to CI after PLR (r=0.88, p<0.001) (figure 5).

**Table 2:** Baseline values of Cardiac Index, stroke volume and systemic vascular resistance of ED patients presenting with uncomplicated sepsis stratified by their response (>15% increase) in Cardiac Index after a passive leg raise (n=30)

	Responders (n=7)	Non-responders (n=23)	P value
Median (IQR)			
Baseline CI (L/min/m^2^)	3.3 (1.9–3.8)	3.3 (2.7–4.0)	0.40
Baseline SV (mL)	63 (44–69)	61 (49–80)	0.73
Baseline SVR (dyn.s/cm^5^)	800 (750–1354)	816 (719–1095)	0.88

SV stroke volume; CI, Cardiac Index; SVR, systemic vascular resistance.

## Discussion

In this study, we found that a (standardised) fluid bolus in patients with sepsis presenting to the ED did not increase (non-invasively measured) CI in the majority of the patients. Surprisingly, we found that only 23% of the ED patients demonstrated a >15% increase in CI, which is generally accepted as a cut-off value for a clinically relevant change.^16^ Responsiveness to fluid, as measured by CI, was strongly correlated with a PLR performed prior to the fluid bolus. However, static measurements of cardiac indices were not predictive of fluid responsiveness.

Fluid therapy for sepsis has become more restrictive over the recent years after publication of the ProMISE, ARISE, Process and FEAST trials that showed no benefit of early goal directed therapy,^19–21^ or even potential harm of fluid bolus administration.^22^ However, to date most guidelines^23 24^ still recommend early intravenous fluid administration to hypotensive sepsis patients to correct overt hypovolemia under the presumption that this may improve CO, oxygen delivery and organ function. In these guidelines, the administration of an initial intravenous crystalloid bolus of up to 30 mL/kg of body weight is recommended as a reasonable first step in the haemodynamic management of an adult patient with septic shock.^24^ These guidelines, however, do not provide clear recommendations for patients with sepsis who are not in shock: Although some guidelines mention that intravenous fluids should be considered, it is not mentioned when, nor how much fluids should be administered.

Our findings demonstrate that a simulated fluid bolus of around 300 mL does not result in a clinically relevant increase in CI in the majority of patients with sepsis without shock. This could not be explained by the volume of the simulated fluid bolus, as previous studies in other populations have revealed that PLR-induced changes in CO can reliably predict fluid responsiveness regardless of ventilation mode and cardiac rhythm.^25^ An alternative explanation is that increases in preload do not automatically translate in an increase in CI: the relation between preload and CI as represented in the Frank-Starling curve^26^ is modified by the complex interaction of multiple components of the cardiovascular system, such as systemic filling pressure, right atrial pressure, venous resistance, ventricular compliance, cardiac contractility and afterload.^7 9^ PLR may affect many of these components concurrently and some of these even in a negative direction (eg, by increasing right atrial pressure and thereby decreasing venous return). The ultimate effect of PLR on CI is the result of all of these effects, and may vary from person to person. In addition, cardiovascular medication use may modify the relationship between preload and CI. As previous literature has shown that patients with uncomplicated sepsis receive on average 680 mL of intravenous fluids in the first 2 hours of their ED stay,^10^ our findings may indicate that this treatment may not improve haemodynamic status for many of these patients.

In this study, in three out of four patients with sepsis without shock haemodynamic status remained unchanged in response to fluid resuscitation. This is far more than the one in three reported by Leisman *et al* 27 in their study of hypotensive sepsis patients. Unfortunately, we were not able to predict which patients would benefit from fluid administration based on patient characteristics at presentation in the ED. There was a trend for responders to have higher urea and creatinine blood concentrations, but significance was only reached for eGFR. These findings may suggest that hydration status and/or intravascular volume status (ie, point on the Frank starling curve) may be a determinant of the effect of increasing preload on SV (and thereby CO and CI): the more a patient is dehydrated, the more likely a PLR or a fluid bolus is to increase CI by increasing SV.

Fluid responders tended to be older and to have a lower HR. This may indicate that although elderly patients benefit from fluid administration (as they increase their CO), their HR response may be blunted (eg, by pathophysiological changes or medication use). This is in line with a previous study wherein it was demonstrated that fluid requirement is higher for elderly patients with an infection compared with younger patients.^28^ In this regard it is important to mention that the average age (75) of our study population was high compared with other populations.^10 28^ If and how this has affected our study results is difficult to predict, as not only fluid requirement may be higher in the elderly, but fluid responsiveness may as well be related to age (eg, due to the higher prevalence of pre-load dependent comorbidities as atrial fibrillation).

The finding that static measurements of SV, SVR, CO and CI at baseline could not predict fluid responsiveness may well be explained by the fact that these measurements not only reflect fluid status, but also cardiovascular status, including myocardial/valvular function and overall vascular tone. On the contrary, we demonstrate that dynamic fluid status assessment with PLR can be used as a reliable predictor to test which patients presenting with sepsis in the ED would benefit from fluid administration, as the correlation between changes in CI between PLR and fluid bolus (500 mL NaCl 0.9%) was high (r=0.88). Non-invasive CO monitoring in combination with PLR may therefore be a valuable new tool to help the clinician tailor sepsis treatment to individual patients, especially in elderly patients. This is of increasing importance as the prevalence of sepsis is rising,^29^ and the average sepsis patient presenting in the ED of most western countries is not only getting older, but also has more comorbidities requiring careful consideration of all treatment options.

Our study has several important limitations. First, although we compared patient, disease and treatment characteristics of responders and non-responders to PLR, some variables (such as fluid intake (and administration) in the days prior to ED presentation, cardiovascular comorbidities (especially LVF and RVF, both in systole and diastole) and cardiovascular medication use (especially beta-blocking drugs)) were not recorded. As fluid responsiveness to PLR is dependent both on preload and cardiac function, our findings may have been influenced by comorbidities and/or cardiovascular medication use. Second, our study carries the potential of sampling bias, as we conducted our study in a convenience sample of ED patients with sepsis but not septic shock. Shift hours (night-time vs daytime), ED crowding and other factors might have influenced patient selection and thereby outcome, which affects generalisability of our results. Generalisability is further affected by inclusion criteria, as we relied on temperature <36 or >38°C without an obvious cause for the hypothermia or hyperthermia to define the presence of an infection. Third, since no point-to-point individual data were available from previous studies, our sample size calculation was based on the overall response rather than our predefined individual primary endpoint. In addition, our study lacks a control arm and therefore we cannot rule out a change in CI in the absence of PLR or fluid bolus. Finally, the number of patients that had to be excluded due to an inability to either fit the finger cuff (1) or obtain a reliable signal (6) to measure CI non-invasively was substantial and may have resulted in selection bias. Although the Clearsight technology has been shown to be a reasonable alternative to invasive monitoring for the assessment of trends in CO,^12 13^ one has to obtain a reliable signal first. This may also limit the practical use of non-invasive output measurements to guide fluid therapy in patients presenting in the ED with uncomplicated sepsis.

### Conclusion

The results of the present study demonstrate that in patients with sepsis in the absence of shock, three out of four patients are unresponsive to a standardised fluid challenge. Non-invasive CO monitoring in combination with a PLR test has the potential to identify patients who might benefit from fluid resuscitation and may contribute to better tailored treatment.

## Data Availability

Deidentified participant data and additional information (study protocol) are available upon reasonable request from the corresponding author.

## References

[1] Burki TK . Sharp rise in sepsis deaths in the UK. Lancet Respir Med 2018;6:826–5. 10.1016/S2213-2600(18)30382-5 30219243

[2] Rhee C , Jones TM , Hamad Y , et al. Prevalence, underlying causes, and preventability of sepsis-associated mortality in US acute care hospitals. JAMA Netw Open 2019;2:e187571. 10.1001/jamanetworkopen.2018.7571 30768188 PMC6484603

[3] Sepsis Alliance. Sepsis fact sheet. Available: https://www.sepsis.org/downloads/2016_sepsis_facts_media.pdf [Accessed January 6th, 2020].

[4] Singer M , Deutschman CS , Seymour CW , et al. The third International consensus definitions for sepsis and septic shock (Sepsis-3). JAMA 2016;315:801–10. 10.1001/jama.2016.0287 26903338 PMC4968574

[5] Rhodes A , Evans LE , Alhazzani W , et al. Surviving sepsis campaign. Crit Care Med 2017;45:486–552. 10.1097/CCM.0000000000002255 28098591

[6] Brown RM , Semler MW . Fluid management in sepsis. J Intensive Care Med 2019;34:364–73. 10.1177/0885066618784861 29986619 PMC6532631

[7] Marik P , Bellomo R . A rational approach to fluid therapy in sepsis. Br J Anaesth 2016;116:339–49. 10.1093/bja/aev349 26507493

[8] Ueyama H , Kiyonaka S . Predicting the need for fluid Therapy—Does fluid responsiveness work? j intensive care 2017;5:34. 10.1186/s40560-017-0210-7 28603624 PMC5461727

[9] Dombrovskiy VY , Martin AA , Sunderram J , et al. Rapid increase in hospitalization and mortality rates for severe sepsis in the United States: a trend analysis from 1993 to 2003*. Crit Care Med 2007;35:1244–50. 10.1097/01.CCM.0000261890.41311.E9 17414736

[10] ter Avest E , de Jong M , Brűmmer I , et al. Outcome predictors of uncomplicated sepsis. Int J Emerg Med 2013;6:9. 10.1186/1865-1380-6-9 23566350 PMC3637101

[11] Shapiro NI , Wolfe RE , Moore RB , et al. Mortality in emergency department sepsis (MEDS) score: a prospectively derived and validated clinical prediction rule*. Crit Care Med 2003;31:670–5. 10.1097/01.CCM.0000054867.01688.D1 12626967

[12] Fischer MO , Avram R , Cârjaliu I , et al. Non-Invasive continuous arterial pressure and cardiac index monitoring with Nexfin after cardiac surgery. Br J Anaesth 2012;109:514–21. 10.1093/bja/aes215 22750726

[13] Pour-Ghaz I , Manolukas T , Foray N , et al. Accuracy of non-invasive and minimally invasive hemodynamic monitoring: where do we stand? Ann Transl Med 2019;7:421. 10.21037/atm.2019.07.06 31660320 PMC6787372

[14] Jabot J , Teboul J-L , Richard C , et al. Passive leg raising for predicting fluid responsiveness: importance of the postural change. Intensive Care Med 2009;35:85–90. 10.1007/s00134-008-1293-3 18795254

[15] Cherpanath TG , Hirsch A , Geerts BF . Predicting fluid responsiveness by passive leg raising: a systematic review and meta-analysis of 23 clinical trials. Crit Care Med 2016;44:981–91. 26741579 10.1097/CCM.0000000000001556

[16] William T , Young C , John A . Edwards clinical education quick guide to cardiopulmonary care. 4 edn, 2018.

[17] Bubenek S , Craciun M , Miclea I . Noninvasive continuous cardiac output by the Nexfin before and after preload-modifying maneuvers: a comparison with intermittent Thermodilution cardiac output. Anesthesia & Analgesia 2013;117:366–72. 23757471 10.1213/ANE.0b013e31829562c3

[18] von Elm E , Altman DG , Egger M , et al. The strengthening the reporting of observational studies in epidemiology (STROBE) statement: guidelines for reporting observational studies. The Lancet 2007;370:1453–7. 10.1016/S0140-6736(07)61602-X 18064739

[19] Mouncey PR , Osborn TM , Power GS , et al. Trial of early, goal-directed resuscitation for septic shock. N Engl J Med 2015;372:1301–11. 10.1056/NEJMoa1500896 25776532

[20] Peake SL , et al, ARISE Investigators. Goal-Directed resuscitation for patients with early septic shock. N Engl J Med 2014;371:1496–506. 10.1056/NEJMoa1404380 25272316

[21] Yealy DM , Kellum JA , et al. A randomized trial of protocol-based care for early septic shock. N Engl J Med 2014;370:1683–93. 10.1056/NEJMoa1401602 24635773 PMC4101700

[22] Maitland K , Kiguli S , Opoka RO , et al. Mortality after fluid bolus in African children with severe infection. N Engl J Med 2011;364:2483–95. 10.1056/NEJMoa1101549 21615299

[23] NICE. Sepsis: recognition, diagnosis and early management. NG51 2016.

[24] Levy MM , Evans LE , Rhodes A . The surviving sepsis campaign bundle: 2018 update. Intensive Care Med 2018;44:925–8. 10.1007/s00134-018-5085-0 29675566

[25] Cavallaro F , Sandroni C , Marano C , et al. Diagnostic accuracy of passive leg raising for prediction of fluid responsiveness in adults: systematic review and meta-analysis of clinical studies. Intensive Care Med 2010;36:1475–83. 10.1007/s00134-010-1929-y 20502865

[26] Patterson SW , Starling EH . On the mechanical factors which determine the output of the ventricles. J Physiol 1914;48:357–79. 10.1113/jphysiol.1914.sp001669 16993262 PMC1420422

[27] Leisman DE , Doerfler ME , Schneider SM , et al. Predictors, prevalence, and outcomes of early crystalloid responsiveness among initially hypotensive patients with sepsis and septic Shock*. Crit Care Med 2018;46:189–98. 10.1097/CCM.0000000000002834 29112081

[28] Ko SY , Esteve Cuevas LM , Willeboer M , et al. The association between intravenous fluid resuscitation and mortality in older emergency department patients with suspected infection. Int J Emerg Med 2019;12:1. 10.1186/s12245-018-0219-2 31179911 PMC6326108

[29] Hajj J , Blaine N , Salavaci J . The “Centrality of Sepsis”: A Review on Incidence, Mortality, and Cost of Care. Health Care 2018;6:90. 10.3390/healthcare6030090 PMC6164723 30061497

