# Supplementary figures and images for: Non-invasive assessment of fluid responsiveness to guide fluid therapy in patients with sepsis in the emergency department: a prospective cohort study
Source: Emerg Med J. 2021 Apr 22;38(6):416–22. doi: 10.1136/emermed-2020-209771 (PMC8165141; doi:10.1136/emermed-2020-209771)

**Figure 1.** 45 degrees angled ramp for performing the PLR

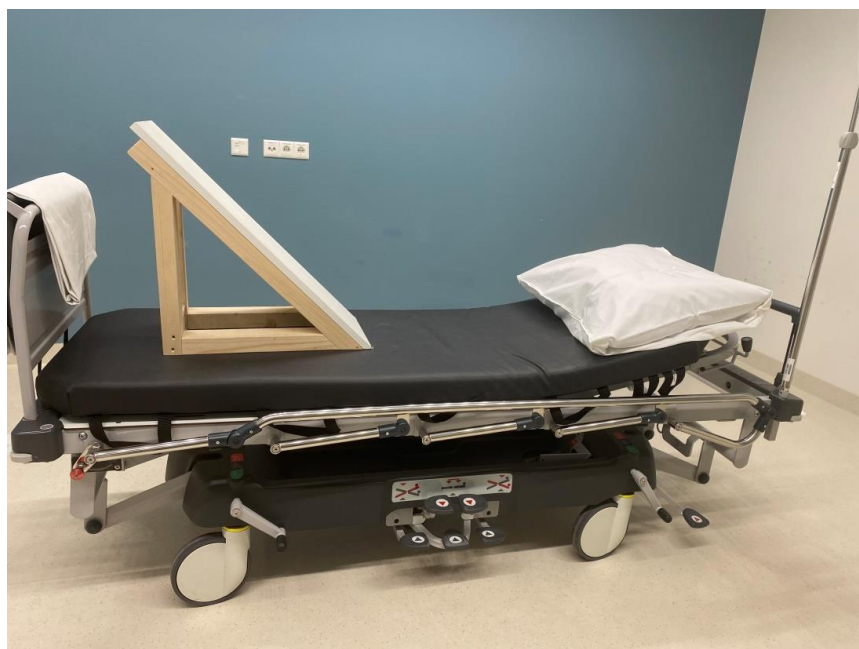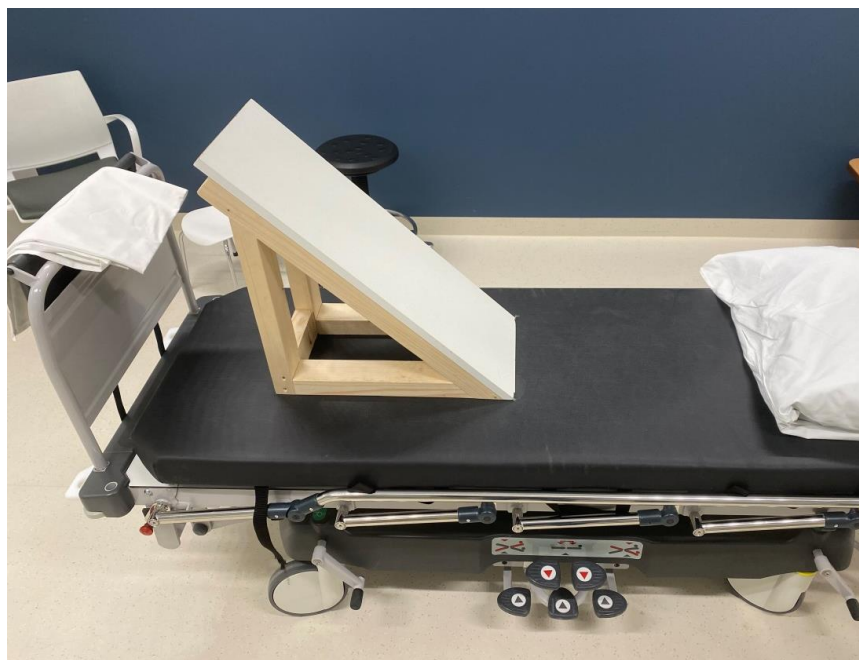

Supplement: Supplementary data [file emermed-2020-209771supp001.pdf]
