# Supplementary material for: Non-invasive assessment of fluid responsiveness to guide fluid therapy in patients with sepsis in the emergency department: a prospective cohort study
Source: Emerg Med J. 2021 Apr 22;38(6):416–22. doi: 10.1136/emermed-2020-209771 (PMC8165141; doi:10.1136/emermed-2020-209771)
Supplement: Supplementary data [file emermed-2020-209771supp002.pdf]

Table 2. *Patient- and treatment characteristics of ED patients presenting with uncomplicated sepsis stratified by their response (>15% increase)in cardiac index after a passive leg raise (n=30).*

	Responders (n=7)	Non- responders (n=23)	
**Median [IQR] or n (%)**			*P*-value
**Demographics**			
Gender (male)	4 (57.1)	14 (60.9)	1.00
Age (years)	82 [73-85]	73 [68-83]	0.19
Length (cm)	170 [156–182]	173 [167-18]	0.45
Weight (kg)	72 [65-82]	78 [70-90]	0.64
**Prehospital**			
Duration of symptoms (days)	2 [1-3]	1[1-5]	0.79
Antibiotic therapy started (n,%)	2 (28.6)	5 (21.7)	1.00
Fluid therapy started	0 (0)	4 (18.2)	0.55
Amount of fluid		375 [200-500]	
Oxygen therapy started	3 (42.9)	10 (43.5)	1.00
**Vital signs at presentation**			
Heartrate (bpm)	96 [91-115]	110 [103-115]	0.28
SBP (mm Hg)	128 [117-180]	130 [115-153]	0.79
MAP (mmHg)	96 [71-107]	88 [78-110]	0.96
SpO_2 (%)	93 [93-94]	94 [93-97]	0.50
RR (/min)	28 [22-32]	25 [22-30]	0.51
Temperature (°C)	39.4 [38.8-39.8]	38.9 [38.5-39.5]	0.57
AVPU (n,%)			
A	6 (85.7)	18 (78.3)	1.00
V	1 (14.3)	5 (21.7)	
P/U	0	0	
GCS	15 [14-15]	15 [14-15]	0.69
Confusion present (n,%)	2 (28.6)	9 (39.1)	1.00
**Laboratory results**			
POCT bloodgas *			
Venous	6 (85.7]	21 (91.3)	0.57
Arterial	1 (14.3]	1 (4.4)	
pH	7.43 [7.42-7.47]	7.44 [7.42-7.45]	0.94
pCO_2 (kPA)	4.3 [3.3-7.9]	4.9 [3.8-6.9]	0.78
Bicarbonate (mmol/L)	28 [27-31]	26 [24-28]	0.036
Lactacte (mmol/L)	1.4 [0.9-1.7]	1.6 [1.1-2.2]	0.57
Hb (mmol/L)	7.4 [6.6-8.9]	8.4 [7.6-9.5]	0.15
WBC (10^9/L) (4–10)	17.3 [6.7-18.7]	14.7 [11.9-18.2]	1.00
CRP (mg/L) (<5)	72 [54-184]	99 [28-267]	0.75
Platelets (10^9/L) (150-400)	194 [151-259]	239 [148-319]	0.52
Creatinine (umol/L)	165 [102-185]	98 [74–136]	0.070
eGFR / 1.73m^2	37 [23-47]	64 [44-78]	0.009
BUN (mmol/L) (2.5-7.5)	12.5 [8.9-14.4]	7.8 [5.9-11.9]	0.070
Bilirubin (umol/L) (<17)**	11 [5-16]	13 [9-15]	0.39

**Presumed source of infection(s) (n,%)**			1.00
Pulmonary	3 (42.9)	9 (39.1)	
Urinary	4 (57.1)	10 (43.5)	
Abdominal	0 (0)	3 (13.0)	
CNS	0 (0)	0 (0)	
Skin	0 (0)	1 (4.4)	
Other	0 (0)	1 (4.4)	
**Total amount of fluid in ED**	600 (520- 1820)	900 (520-1020)	0.86
**Sepsis warning and severity scores**			
SIRS	3 (3-4)	4 (3-4)	0.36
NEWS	8 (6-9)	8 (7-9)	0.94
MEWS	5 (3-6)	5 (4-7)	0.42
qSOFA	1 (1-2)	1 (1-2)	0.70
CURB-65	3 (2-3)	2 (1-3)	0.19
**Disposition (n,%)**			
Normal ward	7(100)	23 (100)	
ICU	0 (0)	0 (0)	-
Re-disposition to ICU within 48 hours	0 (0)	0 (0)	-
**Outcome (n,%)**			
30 days mortality	0 (0)	1 (4.4)	1.00

Legend table 2: SBP, systolic blood pressure; MAP, mean arterial pressure; SpO_2, oxygen saturation; RR respiratory rate; AVPU, Alert-Verbal-Painful-Unresponsive; GCS, Glasgow Coma Scale; SIRS, systemic inflammatory response; NEWS,national early warning score; MEWS, modified early warning score; qSOFA, quick sepsis related organ failure assessment; CURB-65, pneumonia severity score. *ABGA/VBGA data were obtained for 29 patients, ** bilirubin data were obtained for 27 patients
